# Digital home-based physical activity promotion for older adults after total hip arthroplasty: protocol for the randomized controlled iPATH trial

**DOI:** 10.1186/s12877-026-07013-9

**Published:** 2026-01-24

**Authors:** Theresa Buchner, Sarah Knopf, Ann-Sophie Haas, Tobias Eckert, Rebecca Fritz, Marie Merling, Anja Sander, Christina Klose, Tilman Walker, Jürgen M. Bauer, Clemens Becker, Christian Werner, Tobias Reiner

**Affiliations:** 1 Geriatric Center, Medical Faculty Heidelberg, Heidelberg University, Heidelberg, Germany; 2 Department of Orthopaedics, Heidelberg University Hospital, Heidelberg, Germany; 3 Institute of Medical Biometry, Heidelberg University, Heidelberg, Germany

**Keywords:** Hip replacement, Postoperative care, Telerehabilitation, Exercise, Patient education, Sedentary behavior, Ambulatory monitoring, Mobility limitation

## Abstract

**Background:**

Total hip arthroplasty (THA) effectively alleviates pain and improves physical function. However, an increase in patients’ physical activity (PA) is often not observed after surgery. This paradox may result from short rehabilitation periods and persistent sedentary habits and be further influenced by methodological limitations of previous PA monitoring approaches. Digital home-based exercise programs and personal caching (PC) using behavioral change techniques for PA promotion, together with recent advances in PA monitoring, may offer new opportunities to address these challenges. The aim of the iPATH study is to evaluate the efficacy of two novel post-rehabilitation interventions, consisting of a digital home-based physical exercise program with or without PC, compared with usual care, for increasing PA in older adults following THA using advanced PA monitoring approaches.

**Methods:**

In this monocentric, three-arm randomized controlled trial, 213 older THA patients (≥ 65 years) will be assigned in a 1:1:1 ratio post-rehabilitation to a 12-week digital home-based exercise program (“Keep On Keep Up”) with or without additional PC for PA promotion, or usual care. The primary outcome is the mean daily step count at six months post-THA; mean daily walking distance serves as a subordinated primary outcome. Both outcomes will be collected preoperatively, post-rehabilitation, and at six months postoperatively using a body-fixed inertial measurement unit (AX6, Axivity Ltd.) combined with newly validated processing algorithms (Mobilise-D computational pipeline). Secondary outcomes include other digital and self-reported mobility outcomes, physical capacity, hip pain and function, psychological factors, falls, intervention acceptability, and health-related resource use. Primary analyses will follow the intention-to-treat principle.

**Discussion:**

The digital home-based interventions are expected to increase PA compared with usual care. If effective, they have the potential to enhance patient health, reduce morbidity and mortality risk, and be implemented as routine post-rehabilitation care for older adults recovering from THA.

**Trial registration:**

ClinicalTrials.gov (NCT07135843); prospectively registered on August 22, 2025.

**Supplementary Information:**

The online version contains supplementary material available at 10.1186/s12877-026-07013-9.

## Background

Hip osteoarthritis is a leading cause of pain, reduced quality of life and inactivity in older adults. Total hip arthroplasty (THA) is an effective treatment for hip osteoarthritis, alleviating pain and improving physical function [1–3]. Despite these benefits, low levels of preoperative physical activity (PA) often persist after THA. Evidence from systematic reviews and meta-analyses indicates no or only modest increases in PA following THA, with many patients remaining less active than healthy controls during the first postoperative year [4–8]. Low levels of PA are associated with increased morbidity, mortality, and healthcare costs [9–11]. Consequently, the persistence of low PA after THA represents a missed opportunity to improve long-term health outcomes in this patient population.

Postoperative rehabilitation following THA typically consists of inpatient or outpatient programs lasting approximately three weeks [12]. These programs primarily target pain reduction and functional recovery, but may not be specifically designed or primarily intended to promote long-term behavior change or reduce sedentary behavior. This may partly be attributable to the short duration of standard rehabilitation, as habit formation typically requires several months [13]. In addition, compared with healthy individuals, patients undergoing THA and standard rehabilitation continue to exhibit deficits in physical function for up to two years postoperatively [1, 14–16], suggesting that the duration of standard rehabilitation may also be insufficient to fully restore long-term physical function. Evidence from exercise studies in older adults indicates that training durations of several months are typically needed to produce meaningful effects, whereas shorter durations are less effective [17–20]. Extending postoperative rehabilitation to such durations, however, remains largely unfeasible within existing healthcare structures for health-economic and societal reasons.

Self-administered home-based exercise programs may represent a promising approach to support functional recovery after THA, particularly when supported by digital tools [21–23]. In patients undergoing THA, unsupervised home-based exercise programs have been shown to be safe and non-inferior to formal supervised programs delivered in outpatient settings and may therefore constitute a cost-effective strategy for post-rehabilitation care following THA [24, 25]. Digital technologies may facilitate the feasibility, implementation, and effectiveness of such home-based exercise programs by improving accessibility, supporting adherence and motivation through interactive features, and promoting exercise quality through interactive, video-based guidance and training plans [22]. However, evidence-based digital home-based exercise programs alone may be insufficient to sustainably promote habitual PA behavior in daily life among older adults [26].

Personal coaching (PC), a patient-centered process in which a coach supports individuals in changing lifestyle-related behaviors using behavior change techniques (BCTs; e.g., goal setting, self-monitoring, feedback, barrier identification, and problem solving) [27], may offer an opportunity to promote PA among patients following THA. Systematic reviews and meta-analyses have shown that PC incorporating BCTs are effective in increasing PA among older adults [28], persons with physical disabilities [29], and individuals with chronic conditions [30, 31]. However, evidence for the effectiveness of BCTs in promoting PA following rehabilitation for THA is scarce. Previous studies are restricted to case series [32] and a randomized controlled trial (RCT) including mixed lower-limb arthroplasty populations (total hip and knee arthroplasty) and employing only a limited number of BCTs (goal setting, self-monitoring, feedback) [33]. To our knowledge, no methodologically rigorous RCT has yet evaluated the efficacy of PC incorporating multiple BCTs to promote PA in the post-rehabilitation phase among older adults following THA.

Prior research has investigated changes in objective measured PA following THA primarily using basic amount-related PA parameters, such as step count or walking and active duration [4–8]. Recent advancements in sensor technology, combined with newly developed data processing algorithms, now enable valid measurement of a wide range of PA parameters in real-world settings, so-called digital mobility outcomes (DMOs), in both clinical and non-clinical older populations [34, 35]. These DMOs encompass not only the amount of walking (e.g., step count, walking duration), but also walking pattern (e.g., number of walking bouts [WB], WB duration), pace (e.g., walking speed, stride length), rhythm (e.g., cadence, stride duration), and variability (e.g., bout-to-bout variability in walking speed).

The aim of this study is to evaluate the efficacy of two novel post-rehabilitation interventions consisting of a 12-week digital home-based physical exercise program, with or without additional PC, compared with usual care, for increasing PA six months postoperatively among older adults following THA, using advanced PA monitoring approaches. We hypothesize that both interventions will result in greater increases in PA than usual care, with the intervention including PC leading to the greatest improvements.

## Methods

### Study design and setting

**Fig. 1:**
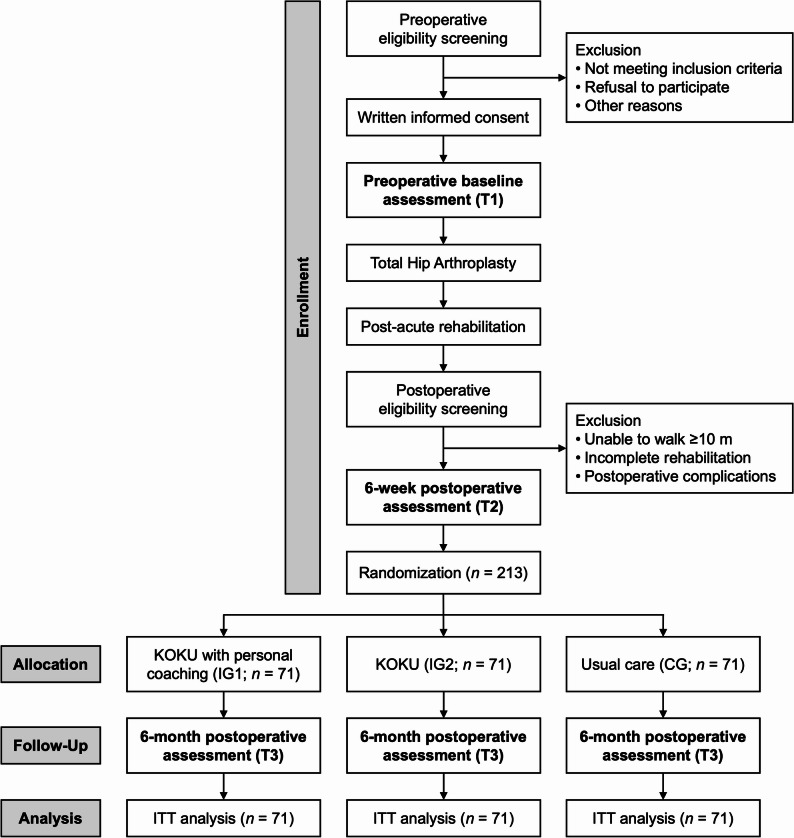
Flowchart of the iPATH study. Abbreviations: *KOKU* Keep On Keep Up, *ITT* Intention-to-treat

The iPATH study (“Digital home-based Physical Activity promotion for older adults after Total Hip arthroplasty”) is a three-arm, assessor-blinded, randomized, parallel-group, monocentric, superiority trial with a 12-week intervention period. Outcomes are assessed 1 week preoperatively (T1), 6 + 2 weeks postoperatively (T2), and 6 months postoperatively (T3; 26 ± 2 weeks). Participant recruitment and on-site outcome assessments will take place at the Department of Orthopaedics, Heidelberg University Hospital, Heidelberg, Germany. Participants will be randomly allocated after T2 to one of two intervention groups (IG) or the control group (CG). The digital interventions will be delivered remotely and implemented at participants’ homes, starting 8 ± 2 weeks after THA following completion of the standard rehabilitation phase. The study flowchart is shown in Fig. 1. The trial was preregistered at ClinicalTrials.gov (NCT07135843) on August 22, 2025. This protocol is reported in accordance with the Standard Protocol Items: Recommendations for Interventional Trials (SPIRIT) guidelines [36].

### Eligibility criteria and recruitment

Community-dwelling older adults (≥ 65 years) undergoing THA are eligible for inclusion if they are able to walk independently (with or without a walking aid), have internet (WiFi) access at home, and have no visual or hearing impairments, cognitive impairment, pending or incomplete rehabilitation, or severe comorbidities or complications that would preclude participation in an exercise program. An overview of all eligibility criteria is provided in Table 1.

**Table 1:** Eligibility criteria for study participation

Preoperative screening		Postoperative screening (4 weeks post-THA)
Inclusion criteria	Exclusion criteria	Exclusion criteria
• Age ≥ 65 years • Coxarthrosis with indication for THA • Residence within a radius of ≤ 50 km from Heidelberg • Ability to walk ≥ 10 m (with/without walking aid) • Access to the internet (WiFi) at home • Written informed consent	• Insufficient German language skills • Insufficient hearing to communicate via (video) telephone • Insufficient vision to recognize study materials • Contralateral coxarthrosis with indication for THA • Severe medical condition that compromises physical fitness (heart failure NYHA ≥ 3, heart valve defects associated with syncope, cardiac arrhythmia with dizziness, COPD with oxygen therapy, cancer with chemotherapy and/or radiation, Parkinson’s disease with rollator use [Hoehn & Yahr stage ≥ 4]) • Living in a nursing home • Planned major medical procedure within the next 6 months with inpatient hospital stay (e.g., further surgery) • Simultaneous participation in another competing study • Cognitive impairment (6CIT ≥ 7 points)	• Ability to walk < 10 m (with/without walking aid) • Post-acute rehabilitation after 6-week postoperative assessment (T2) still pending / not yet completed • Postoperative complications that preclude initiation of the digital exercise program within the next 4 weeks

Abbreviations: *THA* Total hip arthroplasty, *NYHA* New York Heart Association, *COPD* Chronic obstructive pulmonary disease, *6CIT* 6-item Cognitive Impairment Test

Potential participants will be consecutively recruited during routine consultations at the Department of Orthopaedics, Heidelberg University Hospital, Heidelberg, Germany. All patients attending the outpatient clinic will be systematically screened for eligibility using a standardized checklist administered by the secretarial staff. The primary reason for ineligibility will be documented.

Following the initial screening, the study team will verify and complete the eligibility assessment, including cognitive screening using the Six-Item Cognitive Impairment Test [37], and assessment for presence of contralateral coxarthrosis requiring THA. Patients with bilateral coxarthrosis requiring THA on both sides will be placed on a waiting list, and participation may be reconsidered at subsequent surgery if a minimum interval of three months between procedures is met. Patients with coxarthrosis without a current indication for THA will likewise be placed on the waiting list and may be re-screened and enrolled later if a surgical indication arises.

Eligible patients will receive detailed verbal and written information about the study objectives, procedures, potential benefits, and risks. Written informed consent will be obtained preoperatively (T1) by medical staff, following renewed explanation and an adequate reflection period. Formal study inclusion and the T1 assessment will proceed only after informed consent has been obtained.

Approximately four weeks after THA, a postoperative screening will be conducted via telephone to confirm continued study eligibility by verifying ability to walk 10 m with or without walking aid, completion of post-acute rehabilitation, and the absence of postoperative complications that would preclude initiation of the digital exercise program within the following four weeks (Table 1). Only participants who maintain eligibility will proceed to the 6-week postoperative assessment (T2) and randomization.

### Participant timeline

Eligible participants will complete the preoperative assessment (T1) one week prior to surgery, aligned with their routine preoperative medical consultation. Following THA and standard postoperative rehabilitation (about 3 weeks), participants who remain eligible will undergo the postoperative assessment (T2) at 6 + 2 weeks after THA. After T2, participants will be randomized to the two IGs or the CG. Following randomization, a one-time home visit (approximately 60 min) will be conducted, during which all participants will receive a tablet device with an introduction to its use, and study-arm specific instructions will be provided. During the 12-week intervention period, participants in the IGs will receive their respective intervention components, whereas the CG will receive usual care. Six months (± 2 weeks) after THA, the final assessment (T3) will take place. All assessments will be conducted on site at the Orthopaedic Clinic Heidelberg and will last approximately 1–1.5 h each. A schematic overview of participant flow, enrollment, intervention, and assessment time points is provided in the study flowchart (Fig. 1) and the participant timeline (Supplementary file 1).

### Interventions

#### Digital home-based exercise program: keep on keep up

Keep On Keep Up (KOKU) is a tablet-based, progressively structured strength and balance exercise program specifically designed for older adults at risk of falls [38]. It comprises 26 different strength and balance exercises based on the Otago Exercise Program [39], and the Fitness and Mobility Exercise (FaME) Program [40], which have been shown to improve physical capacity, reduce falls, and increase self-reported PA in older adults [41, 42]. Each exercise is accompanied by predefined goals and safety instructions displayed on the tablet and is demonstrated by an interactive virtual trainer avatar with audio guidance.

Participants allocated to the IGs will receive instructions in the use of the KOKU app during the home visit. Participants will be encouraged to use KOKU three times per week for approximately 15 min per session over the 12-week intervention period. After the intervention period, participants in the intervention groups may continue using KOKU indefinitely.

Each training session consists of three exercises. Progression to more challenging exercises occurs after a minimum of two weeks and is based on user feedback on perceived physical exertion and safety following each session. If both are rated as adequate, participants advance automatically to the next difficulty level (up to six levels), which is maintained for another minimum of two weeks.

After each exercise, participants are prompted to record the number of repetitions performed. The KOKU app displays the number of completed training sessions and the remaining sessions required to complete the 12-week intervention. In addition to the structured training plan, participants may select additional exercises at any time from the pool of the 26 exercises. If KOKU is not used for two days, the app automatically generates a push notification reminder on the participant’s tablet device.

KOKU has recently been shown to be effective in improving functional outcomes, such as balance and lower-limb strength, in older adults [43]. A German-language version of KOKU has been evaluated in the SMART-AGE [44], where it showed excellent user experience, with no serious adverse events related to its use among community-dwelling older adults [45].

#### Personal coaching

The PC intervention for PA promotion will comprise three tablet-based video call sessions, each lasting about 45–60 min and conducted by trained physiotherapists or sports scientists. The PC is grounded in the principles of self-determination theory (SDT) [46], and integrates evidence-based BCTs [47]. The applied BCTs aims to promote the individual’s autonomy, sense of competence, and relatedness, which represent basic psychological needs according to SDT, thereby enabling participants to integrate PA in their daily routines. Key BCTs will include: (1) provision of information on the benefits of PA, (2) activity-related goal setting, (3) self-monitoring of PA, (4) feedback on PA behavior and related outcomes, (5) action planning, (6) use of prompts and cues for PA, (7) identification and management of PA-related barriers, (8) planning social support for PA, and (9) motivational interviewing (MI). BCTs 1–8, which aim to modulate personal beliefs and PA behavior, are embedded within the communication framework of motivational interviewing [48]. In accordance with this approach, participants and coaches interact on equal terms, reflected in a collaborative and informative communication style.

The PC sessions will be conceptually interconnected, with the BCTs implemented hierarchically across the three video calls. The first session will be conducted two weeks after initiation of KOKU, following the home visit. Based on participants’ previous and current experiences with PA during rehabilitation, they will be encouraged to set individually relevant activity-related goals and to develop action plans to implement these goals. Participants will be offered the option to self-monitor their PA and progress using a simple wrist-worn activity tracker (AS80, Beurer GmbH, Ulm, Deutschland), which they will receive during the home visit along with an introduction it its use and the associated app pre-installed on the tablet. This will allow participants to receive immediate feedback on their PA behavior.

Supplementary file 2 provides a detailed description of both interventions using the Template for Intervention Description and Replication (TIDieR) checklist [49].

### Control

Participants allocated to the CG will receive usual care, consisting of standard health and aged care services routinely available to older adults in the German healthcare systems. In addition, during the home visit, participants will be also provided with a tablet (with an introduction to its use) but without access to the KOKU app and without any PC sessions during the subsequent intervention period.

### Outcomes

An overview of primary, secondary, safety outcomes and descriptive measures are provided in Table 2. Data on all outcomes will be collected at the predefined time points according to the schedule of assessments. Except for DMOs, which will be recorded in participants´ home environments using wearable sensor technology, all measurements will be performed at the study center. To ensure optimal methodological rigor and standardization across assessments, all study personnel receive comprehensive training in all screening and assessment procedures prior to the initiation of participant recruitment.

**Table 2:** Overview of primary, secondary, safety outcome and descriptive measures

Variables		T1 (1 week pre-OP)	T2 (6 weeks post-OP)	INT	T3 (6 months post-OP)
Sociodemographics					
DV	Age, sex, education, marital status, living situation, retirement status	X			
Clinical variables					
DV	Functional Comorbidity Index	X			
DV	Body Mass Index	X			X
DV	Charnley Classification	X			
DV	Fall history	X			
DV	Pre-operative hip characteristics (primary diagnosis, operated side, prior operations)	X			
DV	Surgical information (surgical technique, implant specification)		X		
DV	Range of hip motion, leg length discrepancy, gluteal insufficiency	X	X		X
Mobility					
*Real-world digital mobility outcomes* (AX6, Axivity Ltd., Newcastle, UK)					
PO	Step count	X	X		X
PO	Walking distance	X	X		X
SO	Walking duration, WB count, WB duration, walking speed, stride length, stride duration, cadence, walking variability	X	X		X
*Self-reported daily-life mobility*					
SO	Life-Space Assessment	X			X
SO	UCLA Activity Score	X			X
Physical capacity					
SO	6-Minute Walk Test^a^	X	X		X
SO	Timed Up and Go^a^	X	X		X
SO	30-Second Chair Stand Test	X	X		X
SO	4-Meter Gait Speed Test	X	X		X
SO	Global rating of change in self-perceived walking ability		X		X
Pain & hip function					
SO	Oxford Hip Score	X	X		X
DV	Oswestry Disability Index	X	X		X
Psychological factors					
SO	Short Falls Efficacy Scale-International	X	X		X
SO	Conscientiousness dimension – Big Five Inventory-2	X	X		X
SO	Behavioral Regulation in Exercise Questionnaire	X	X		X
DV	Short Scale of Technology Commitment	X			
DV	Patient Health Questionnaire-2	X			X
Executive functioning					
SO	Trail-Making Test A & B	X	X		X
Health status					
SO	EuroQol 5-Dimension 5-Level	X	X		X
SO	EuroQol Visual Analogue Scale	X	X		X
Falls					
SO	Fall calendar		X	X	X
Training adherence					
OPO	Exercise Adherence Rating Scale				X
OPO	Usage data of the training app				X
Intervention acceptability					
OPO	Theoretical Framework of Acceptability Questionnaire				X
OPO	5-point Likert scale on global acceptability				X
Health-related resource use					
OPO	Questionnaire for Health-Related Resource Use in an Elderly Population	X			X
DV	Type of post-acute rehabilitation program		X		
DV	Participation in other aftercare programs		X		X
DV	Usage of another training app		X		X
Intervention delivery costs					
OPO	Personnel, material, and travel expenses			X	
Harms					
SA	Complications & adverse events		X	X	X

Abbreviations: *INT* Intervention, *pre-OP* Preoperatively, *post-OP* Postoperatively, *DV* Descriptive variable, *PO* Primary outcome, *SO* Secondary outcome, *OPO* Other predefined outcome, *SA* Safety outcomes, *WB* Walking bout, *UCLA* University of California, Los Angeles

^a^instrumented with the mTEST³ system [mHealth Technologies srl, Bologna, Italy] to measure spatiotemporal gait parameters (6-Minute Walk Test) and durations of sit-to-walk, walking, turning, and sitting segments (Timed Up and Go)

#### Primary outcomes

The first primary outcome is the mean daily step count, and the subordinated primary outcome is the mean daily walking distance. Both DMOs will be recorded with a small (23.0 × 32.5 × 8.9 mm) and lightweight (11 g), water-resistant 6-axis inertial measurement unit (IMU; AX6, Axivity Ltd., Newcastle upon Tyne, United Kingdom), fixed at the participant’s lower back (L5 vertebra) using self-adhesive fixing foil, over a maximum measurement period of 7 consecutive days subsequently to the on-site assessments. After the measurement period, participants will remove the IMU themselves and return it to the study center in a prepaid, pre-addressed envelope. The IMU will be configured with a tri-axial accelerometer (range ± 8 g, accuracy 1 mg) and a tri-axial gyroscope (range ± 2000 dps, resolution 70 mdps), with a sampling frequency of 100 Hz. Raw data from the IMU will be standardized according to a predefined data structure described elsewhere [50]. The Mobilise-D computational pipeline (available at https://github.com/mobilise-d/mobgap), which consists of validated algorithms to derive DMOs [51–53], will then be used to identify WBs [54] and extract number of steps, duration, walking speed, stride length, cadence, and stride duration for each of these WBs. Walking distance per WB will be calculated by multiplying two validated DMOs: average walking speed × WB duration [55]. Steps and walking distances at the WB level will then be aggregated to daily level and averaged across up to 7 valid measurement days to yield mean daily step count and mean daily walking distance. A valid measurement is defined as ≥ 3 valid measurement days; a valid measurement day is defined with ≥ 12 h/day of wear time during waking hours (7 am – 10 pm), without weekday/weekend restriction [56].

#### Secondary outcomes

Secondary DMOs will be derived from the IMU recordings using also the validated Mobilise-D computational pipeline, applying the same valid-measurement criteria as for the primary outcomes: walking duration; number of WBs (all, > 10 s, > 30 s, > 60 s); WB duration and 90th percentile (P90) in WB duration; walking speed and P90 walking speed in WBs > 10 s and > 30 s; stride length (WBs 10–30 s and > 30 s); stride duration (all WBs and > 30 s); cadence (all WBs and > 30 s) and P90 cadence (WBs > 30 s); and bout-to-bout variability for WB duration (all WBs), walking speed and stride length (WBs > 30 s), cadence and stride duration (all WBs). Detailed definitions and the daily and weekly calculation procedures of these DMOs are provided elsewhere [34].

Self-reported daily-life mobility will be assessed using the Life-Space Assessment [57, 58] and the UCLA Activity Score [59].

Physical capacity will be measured using the 6-Minute Walk Test (6MWT) [60], the Timed Up and Go (TUG) [61], the 30-Second Chair Stand Test, and the 4-Meter Gait Speed Test [62]. The 6MWT and TUG will be instrumented with the mTEST3 system (mHealth Technologies srl, Bologna, Italy), a CE-certified sensor-based motion analysis system, to measure spatiotemporal gait parameters during the 6MWT (e.g., asymmetry, step length) and segments of movements during the TUG (e.g., sit-to-stand, walking, turning, stand-to-sit). Changes in self-perceived walking ability will be assessed with a 7-item Global Rating of Change scale [63].

Hip pain and function will be assessed using the Oxford Hip Score [64]. Back pain will be measured using the Oswestry Disability Index [65, 66].

Psychological factors will be evaluated using the Short Falls Efficacy Scale-International [67], the conscientiousness dimension of the Big Five Inventory-2 [68], and the Behavioral Regulation in Exercise Questionnaire (motivation regulation in PA) [69, 70].

Self-reported health status will be assessed using the EuroQol 5-Dimension 5-Level and the EuroQol Visual Analogue Scale [71].

Falls will be prospectively self-monitored by participants for each week after T2 assessment using fall calendars, which include documentation sheets to record the date, time, and location of the fall, the activity preceding the fall, and any fall-related injuries.

#### Other predefined outcomes

Adherence to the digital home-based exercise program will be assessed using the Exercise Adherence Rating scale [72] and usage data of the KOKU app.

Intervention acceptability will be evaluated using the Theoretical Framework of Acceptability questionnaire [73] and two separate 5-point Likert Scales on the global acceptability of the KOKU app and the PC intervention.

Health-related resource use will be assessed with an adapted version of the Questionnaire for Health-Related Resource Use in an Elderly Population (German: *‘Fragebogen zur Inanspruchnahme medizinischer und nicht-medizinischer Versorgungsleistungen im Alter’*), by use of the healthcare provider perspective, covering direct medical costs of formal healthcare services (e.g., physician visits, therapeutic services, rehabilitation, outpatient procedures, and hospital stays) within the past three months. Resource utilization will be converted into monetary values using standardized national unit costs [74].

Intervention delivery costs will include personnel, material, and travel expenses directly related to the interventions. Personnel costs will be derived from the national average wages of the professional groups involved, based on the time required for intervention implementation.

#### Descriptive variables

Age, sex, education, marital status, living situation, and retirement status will be collected as sociodemographic characteristics.

Clinical information will include comorbidities [75], body mass index, Charnley classification, fall history, preoperative hip characteristics (primary diagnosis, operated side, prior operations), surgical details (technique, implant specification), and hip function measures (range of hip motion, leg length discrepancy, gluteal insufficiency).

Technology acceptance (Short Scale of Technology Commitment; [76]) and depressive symptoms (Patient Health Questionnaire-2; [77, 78]) will be assessed as descriptive psychological factors. Descriptive variables for health-related resource use will include type of post-acute rehabilitation (outpatient, inpatient, or none), participation in other aftercare programs, and use of other training apps.

### Harms and safety monitoring

Postoperative complications occurring between the preoperative baseline assessment (T1) and the 6-week postoperative assessment (T2) will be recorded at the on-site T2 visit.

Adverse events (AEs) will be documented for the period from the 6-week postoperative assessment (T2) to the 6-month assessment (T3), regardless of any causal relationship to the interventions.

These will be recorded by telephone interview conducted by the coaches at the end of the intervention period and at the very end of the on-site 6-month assessment (T3) by assessors. In addition to systematically collected postoperative and AEs at scheduled assessments, interim events spontaneously reported by participants (e.g., via telephone calls) will also be documented as reported.

The severity of complications and AEs will be classified into: (1) mild = transient and easily tolerated events, causing no or minimal interference with activities of daily living (ADLs) and not requiring medical intervention; (2) moderate = events causing noticeable interference with ADLs and/or requiring minimal or noninvasive medical intervention; (3) severe = events resulting in marked limitation of ADLs, requiring medical intervention or hospitalization, including life-threatening events and death.

An independent Data Safety Monitoring Board (DSMB) will oversee participant safety and trial conduct. The DSMB will consist of an orthopedic surgeon, a geriatrician, and a biometrician. It will regularly review accumulating reports of AEs, assess their causal relationship with the interventions and severity classification, and make recommendations on potential modifications or discontinuation of the study. The DSMB will operate under a written charter specifying its roles, responsibilities, and decision-making processes.

### Sample size

The sample size was calculated based on the primary hypothesis that KOKU combined with PC (IG1) will increase mean daily step count (first primary outcome) 6 months postoperatively, compared with usual care (CG). Based on previous observational studies, a mean daily step count of 5,000 ± 3,000 steps for the preoperative PA was assumed [79–83]. An increase of 1,500 steps per day in the IGs was assumed, which has been associated with an approximately 20% reduction in all-cause mortality risk among older adults [84, 85]. Under these assumptions, 64 participants per group are required to achieve a statistical power of 80% at a two-sided significance level of 5% using a *t*-test for independent samples. For the subordinated primary outcome (mean daily walking distance), which will be tested in a hierarchical order, a similar effect size is expected. Accounting for an anticipated dropout rate of 10%, the required sample size increases to 71 participants per group, resulting in a final total sample size of 213 participants.

### Randomization and blinding

After the 6-week postoperative assessment (T2), participants will be randomized to one of three study arms in a 1:1:1 allocation ratio using permuted block sizes, stratified by type of post-acute rehabilitation (outpatient, inpatient, or none). The random allocation sequence will be generated automatically using the web-based tool randomizer.at (Institute for Medical Informatics, Statistics and Documentation, Medical University of Graz, Austria; https://www.randomizer.at). Allocation concealment will be ensured through this centralized, Good Clinical Practice-compliant web-based tool. Randomization will be performed exclusively by authorized study personnel with individual access credentials.

All primary and secondary outcome assessments will be undertaken by assessors blinded to group allocation. Other prespecified outcomes, including training adherence and intervention acceptability, will be collected using self-completed questionnaires provided to participants after the final 6-month postoperative assessment (T3). These will be completed at home and returned to the study center by mail using prepaid envelopes.

Usage data of the KOKU app will be downloaded from the tablets of participant in the IGs by assessors only after completing of the on-site T3 assessment.

AEs will be documented by the unblinded coaches during the intervention period (for participants of both IGs) and at the end of the intervention period (for all participants). At T3, AEs will also be recorded by blinded assessors, but only at the end of the visit, after all other on-site assessments have been completed.

Processing of the DMOs after return of the IMU will be conducted at T3 by blinded study personnel who are not involved in the on-site T3 assessment.

### Data collection and management

The Institute for Medical Biometry, Heidelberg University (IMBI) is responsible for data management within the study. Study data will be collected and managed using Research Electronic Data Capture (REDCap; [86]), a secure, web-based application that supports data entry with role-based access control, audit trails, and integrated data validation. Data transmission is encrypted (Secure Socket Layer, SSL), and access is restricted to authorized users with role-specific permissions. To ensure confidentiality, all data are pseudonymized, and changes are logged with time stamps in an audit file. Data validation rules are predefined in a data validation plan; data completeness, plausibility, and internal consistency are verified at the time of data entry using automated edit checks and additional validation programs that generate queries. Once all queries have been resolved and no further corrections are required, the database will be locked and released for statistical analysis. At the end of the study, data will be exported into reusable formats (e.g., CSV files) and archived by the principal investigator for long-term storage.

### Statistical analysis

For the first primary endpoint, the null hypothesis states that there is no difference in the mean daily step count at 6-months post-THA (T3) between IG1 and CG. The primary analysis will use linear regression at a two-sided significance level of 5%, adjusting for the stratification factor post-acute rehabilitation (outpatient, inpatient, or none) and preoperative mean daily step count. If this hypothesis is rejected, the subordinated primary endpoint (mean daily walking distance) at T3 between IG1 and CG will be tested (adjusted for post-acute rehabilitation and preoperative mean daily walking distance).

Subsequently, the following hypotheses will be tested hierarchically: IG2 (KOKU alone) vs. CG for mean daily step count at T3; IG2 vs. CG for mean daily walking distance at T3; IG1 vs. IG2 for mean daily step count at T3; and IG1 vs. IG2 for mean daily walking distance at T3. If any test in this sequence cannot be rejected, all subsequent tests will be considered exploratory only. This hierarchy reflects the assumption that IG1 (KOKU with PC) is at least as effective as, or superior to, IG2 (KOKU alone).

The primary analysis follows the intention-to-treat principle, with participants analyzed according to their randomized group. Intercurrent events are handled as follows: for non-compliance, additional interventions, AEs after randomization, new illnesses, or falls occurring between T2 and T3, a treatment-policy strategy (ICH E9 (R1)) will be applied, such that the estimated treatment effect includes the impact of these events. Death unrelated to the intervention will be handled under a hypothetical strategy, assuming the intervention does not affect the timing or occurrence of death within the observation window. If the T3 assessment cannot be performed due to a training-related injury or fatal event clearly attributable to the intervention, a “worst outcome” (step count = 0 steps/day, walking distance = 0 m/day) will be imputed as a composite strategy.

Missing values for primary endpoints due to dropout or loss to follow-up will be imputed under a missing-at-random (MAR) assumption using multiple imputations with predictive mean matching. The imputation model includes the stratification factor (post-acute rehabilitation type) and mean daily step count / mean daily walking distance at T1 and T2 for first and subordinated primary endpoints, respectively. Relevant intercurrent events, and other important prognostic variables will also be incorporated.

To investigate the robustness of the results, sensitivity analyses will be conducted, including adjustments for further potential confounders such as age and comorbidity burden, as well as a reference-based approach (“copy reference”) to explore departures from MAR under a missing-not-at-random (MNAR) scenario. The reference-based approach assumes that participants in the IGs with missing data gradually converge toward the outcome trajectory of the CG.

A supplementary estimand will be used to quantify treatment effects under alternative strategies, including hypothetical strategies for non‑compliance and composite strategies (“worst outcome”) for cases in which the T3 assessment cannot be completed for intervention‑related reasons. A predefined subgroup analysis will compare participants with higher vs. lower preoperative PA, as those with higher baseline PA may derive less additional benefit from the interventions.

Secondary endpoints will be analyzed descriptively using appropriate summary statistics and confidence intervals. Safety analyses will summarize absolute and relative frequencies of postoperative complications from randomization onward.

All planned analyses will be described in detail in the Statistical Analysis Plan, which will be finalized prior to database closure and any conduct of unblinded analyses.

### Patient and public involvement

Prior to initiating the interventions, a patient advisory board comprising five individuals who have previously undergone THA will be established to contribute patient perspectives to the study design. Up to five advisory board sessions will be held throughout the study period. During the first meeting, advisory board members will evaluate the usability and applicability of KOKU on a tablet, with a special focus on the suitability of the exercises for older adults following THA. The second meeting will include a critical review of supporting materials (e.g., flyers and manuals), with emphasis on design and comprehensibility, consultation on tablet setup workflows prior to distribution to participants, and evaluation of the study website layout. Before the enrolment of the first participant, selected advisory board members will take part in pilot testing of the assessment procedures and video-call setup for the PC. In the final meeting, advisory board members will co-design strategies for providing individual feedback to participants and will support dissemination of the overall study results to the non-scientific public.

### Protocol amendments

Any substantial protocol amendments will be submitted to the Ethics Committee of the Medical Faculty Heidelberg for re-evaluation and approval, registered at ClinicalTrials.gov, and reported in the primary publication of the study results. Results will be published in peer-reviewed journals and presentations at scientific conferences.

## Discussion

The iPATH study is designed to investigate the efficacy of a post-rehabilitation digital home-based exercise program, with or without PC, for increasing PA in older adults following THA. To our knowledge, this is the first RCT to evaluate a self-administered digital exercise program combined with remote, technology-supported PC, incorporating a broad range of BCTs to promote PA specifically in older adults after THA, and using advanced PA monitoring approaches to comprehensively assess real-world DMOs.

The iPATH study addresses a clinically relevant and persistent challenge in postoperative care for older adults undergoing THA, namely the limited translation of improved physical function and reduced pain into sustained and meaningful increases in habitual PA. This discrepancy highlights a critical gap in current rehabilitation pathways after THA and suggests functional improvements alone may be insufficient to induce health-relevant, long-term behavior change.

Recent meta-analytic evidence in lower-limb arthroplasty patients indicates that interventions specifically designed for PA promotion, particularly those integrating BCTs and wearable activity monitoring, can increase PA, whereas interventions limited to the delivery or prescription of exercise alone shown no significant effects on PA [87].

These findings underscore the potential of PA interventions that combine exercise with evidence-based BCTs, including self-monitoring via activity trackers, to promote in the post-rehabilitation phase among older adults following THA, as implemented in the iPATH study.

A key strength of this study is its clear focus on digital health solutions capable of extending the rehabilitation trajectory beyond the limited duration of standard postoperative rehabilitation following THA. Digital technologies enable the implementation of home-based PA interventions that combine ongoing, flexible access to structured exercise with low-threshold, remotely delivered PC to support sustained engagement in PA and its integration into patients’ daily lives. By reducing common barriers such as transportation constraints, scheduling inflexibility, and reduced access to follow-up care, such approaches may offer a feasible, effective and scalable means of promoting longer-term PA behavior change.

KOKU was selected to implement the digital home-based exercise program for several reasons. First, it combines evidence-based strength and balance exercises for older adults (Otago, FaME) [39, 40] with several BCTs to support exercise adherence [47], such as clear instruction and guidance through avatar-led exercise demonstrations, structured goal setting and action planning via a progressive training plan, self-monitoring through repetition tracking, and prompts delivered via push notification reminders. Second, KOKU has been identified by experts as a high-quality digital application to support the engagement of older adults in performing strength and balance exercises [88]. Third, older users have rated KOKU as engaging, easy to use, and enjoyable after several weeks of independent use, indicating high usability and acceptability in this target population [89]. Finally, the KOKU app complies with European general data protection regulations and is available in German language [45, 90].

The PC intervention adds a theoretically grounded behavioral component that directly addresses motivational and psychological determinants of PA adoption. Older adults following THA may experience motivational challenges related to PA, fear of falling, or low self-efficacy, which can impede the adoption of an active lifestyle despite improvements in physical function [91, 92]. Both systematic reviews of quantitative studies and qualitative syntheses consistently highlight the importance of professional support in promoting PA among older adults [28, 93], supporting the inclusion of PC as a complementary component in the iPATH intervention.

The PC sessions delivered via video calls integrate established BCTs [47] within a self-determination theory framework [46] supported through MI. Remote delivery PA-promoting interventions has emerged as a promising modality, achieving outcomes comparable to face-to-face programs while offering substantial advantages in terms of accessibility and cost-effectiveness [94]. Within the PC intervention, participants will also receive an activity tracker for self-monitoring and feedback, which has been shown to be an effective tool for promoting PA among patients following THA [33, 87, 95]. Together, these components address key determinants, such as personalization, ease of use, and structured feedback, which have been identified as particularly relevant for long-term adherence to technology-support PA interventions among older adults [96].

A further strength of this study is the use of an advanced PA monitoring approach, combining IMUs with recently validated data processing algorithms to capture a broad range of DMOs related to walking amount, pattern, pace, rhythm, and variability. This enables a more detailed and comprehensive picture of PA following THA, which has been lacking in previous studies and extends beyond traditional step counting [6]. The added value of this approach becomes evident when considering that unchanged step counts before and after THA have often been interpreted as indicating no increase in PA. However, this interpretation may be misleading if walking characteristics such as step length or walking speed change over time. Evidence from laboratory-based gait analyses has shown that spatiotemporal gait parameters, including step length and walking speed, typically increase following THA compared with preoperative levels [1]. Consequently, even in the presence of stable step counts, postoperative increases in step length or walking speed may result in greater daily-life walking distances and, therefore, higher overall PA. Until now, spatiotemporal walking parameters could not be validly assessed in real-world settings. The use of recently validated DMOs in the iPATH study enables this type of analysis for the first time among patients following THA.

Several limitations need to be acknowledged. First, the predefined eligibility criteria, which are intended to ensure participant safety during self-administered home-based exercise and to optimize intervention feasibility, may limit the generalizability of the study sample to older adults with substantial frailty, multimorbidity or cognitive and sensory impairments; residents of long-term care facilities; and individuals with limited access to digital infrastructure. Second, the single center recruitment may constrain the external validity of the findings. Third, although assessors will be blinded to group allocation, participants and coaches cannot be blinded, introducing the potential for performance and expectancy bias. This may be particularly relevant in the IG receiving KOKU and PC, where contact time and device feedback will exceed those in the IG receiving only KOKU and the CG. While such differences reflect real-world implementation models, they should be considered when interpreting between-group contrasts. Fourth, the study will not assess longer-term physical activity (PA) trajectories beyond 6 months postoperatively; therefore, the durability of intervention effects beyond this period cannot be determined. Finally, adherence to both KOKU and PC may vary, especially among older adults with competing health or caregiving demands; however, detailed process and usage data will enable exploration of dose-response relationships and identification of implementation challenges.

In summary, the iPATH study is designed to generate robust evidence to inform future post-rehabilitation strategies and clinical guidelines following THA. By evaluating a digital home-based exercise program with and without PC, and by applying an advanced PA monitoring approach, this study will provide novel insights into the effectiveness of technology-supported behaviorally informed interventions for promoting habitual PA after THA. If these interventions prove to be effective, they have the potential to improve health outcomes, attenuate morbidity and mortality associated with physical inactivity and support healthy ageing in the growing population of older adults undergoing THA. Ultimately, the findings may provide a strong rationale for integrating post-rehabilitation digital home-based exercise and coaching approaches into routine post-rehabilitation care pathways following THA.

### Trial status

Participant recruitment for the iPATH study began in September 2025; enrollment of the last participant is planned for July 2027. By the time of submission (January 07, 2026), 19 participants had already been enrolled.

## Supplementary Information

Supplementary Material 1.

Supplementary Material 2.

## Data Availability

No datasets were generated or analysed during the current study.

## Abbreviations

6CIT: 6-item Cognitive Impairment Test
6MWT: 6-Minute Walk Test
ADL: Activity of daily living
AE: Adverse event
BCT: Behavior change technique
CG: Control group
COPD: Chronic obstructive pulmonary disease
DMO: Digital mobility outcome
DSMB: Data Safety Monitoring Board
FaME: Fitness and Mobility Exercise
IG: Intervention group
IMU: Inertial measurement unit
iPATH: Digital home-based Physical Activity promotion for older adults after Total Hip arthroplasty
KOKU: Keep On Keep Up
MAR: Missing-at-random
MNAR: Missing-not-at-random
NYHA: New York Heart Association
P90: 90th percentile
PA: Physical activity
PC: Personal coaching
RCT: Randomized controlled trial
REDCap: Research Electronic Data Capture
SDT: Self-determination theory
SPIRIT: Standard Protocol Items: Recommendations for Interventional Trials
SSL: Secure socket layer
T1: Preoperative assessment
T2: 6-week postoperative assessment
T3: 6-month postoperative assessment
THA: Total hip arthroplasty
TIDieR: Template for Intervention Description and Replication
TUG: Timed Up and Go
WB: Walking bout
WiFi: Wireless fidelity
