# Supplementary material for: Digital home-based physical activity promotion for older adults after total hip arthroplasty: protocol for the randomized controlled iPATH trial
Source: BMC Geriatr. 2026 Jan 24;26:102. doi: 10.1186/s12877-026-07013-9 (PMC12838228; doi:10.1186/s12877-026-07013-9)
Supplement: Supplementary file 1 — Supplementary Material 1. [file 12877_2026_7013_MOESM1_ESM.docx]

**Table S1.** iPATH participant timeline: Schedule of enrollment, interventions, and assessments.

|  | **TRIAL PERIOD** | | | | | | | |
| --- | --- | --- | --- | --- | --- | --- | --- | --- |
|  | Enrollment | | | | | Post-randomization | | Close-out |
| **TIMEPOINT** | **4-6 weeks**  **pre-THA** | **1 week**  **pre-THA** | **THA** | **4-5 weeks**  **post-THA** | **6 weeks**  **post-THA**  (+2 weeks) | **8 weeks**  **post-THA**  (+2 weeks) | **20 weeks**  **post-THA**  (+2 weeks) | **26 weeks**  **post-THA**  (±2 weeks) |
| **ENROLLMENT** | | | | | | | | |
| Pre-surgery eligibility screening | X |  |  |  |  |  |  |  |
| Informed consent |  | X |  |  |  |  |  |  |
| Surgery (THA) |  |  | X |  |  |  |  |  |
| Post-surgery screening |  |  |  | X |  |  |  |  |
| Randomization |  |  |  |  | X |  |  |  |
| **INTERVENTION / COMPARATOR** | | | | | | | | |
| KOKU with personal coaching^a^ |  |  |  |  |  | → | → |  |
| KOKU^a^ |  |  |  |  |  | → | → |  |
| Usual care^a^ |  |  |  |  |  | → | → |  |
| **ASSESSMENTS** | | | | | | | | |
| Sociodemographic characteristics |  | X |  |  |  |  |  |  |
| Clinical characteristics |  | X |  |  | X |  |  | X |
| Mobility |  | X |  |  | X |  |  | X |
| Physical capacity |  | X |  |  | X |  |  | X |
| Pain & hip function |  | X |  |  | X |  |  | X |
| Psychological factors |  | X |  |  | X |  |  | X |
| Executive functioning |  | X |  |  |  |  |  | X |
| Health status |  | X |  |  |  |  |  | X |
| Falls^a^ |  |  |  |  | → | → | → | → |
| Training adherence |  |  |  |  |  |  |  | X |
| Intervention acceptability |  |  |  |  |  |  |  | X |
| Health-related resource use |  | X |  |  | X |  |  | X |
| Intervention delivery costs^a^ |  |  |  |  |  | → | → |  |
| Complications & adverse events |  |  |  |  | X |  | X | X |
| ^a^Arrows indicate continuous delivery of the intervention with concurrent capture of delivery costs, and ongoing documentation of falls.  THA, total hip arthroplasty; KOKU, Keep On Keep Up; PC, personal coaching | | | | | | | | |
