# Supplementary material for: Digital home-based physical activity promotion for older adults after total hip arthroplasty: protocol for the randomized controlled iPATH trial
Source: BMC Geriatr. 2026 Jan 24;26:102. doi: 10.1186/s12877-026-07013-9 (PMC12838228; doi:10.1186/s12877-026-07013-9)
Supplement: Supplementary file 2 — Supplementary Material 2. [file 12877_2026_7013_MOESM2_ESM.docx]

**Table S2** Description of the digital home-based exercise program and the personal coaching using the TIDieR checklist

| **Item** | **Intervention** | |
| --- | --- | --- |
| 1. Brief name | Keep On Keep Up (KOKU) | Personal Coaching (PC) |
| 2. Why | KOKU was selected to extend rehabilitation beyond the limited duration of standard care following THA by providing continued access to a digital home-based exercise program that combines evidence-based strength and balance exercises with BCTs to support training adherence and promote sustained PA. | PC is included to provide structured support from trained staff using evidence-based BCTs to promote sustained PA following THA beyond the digital home-based exercise program. |
| 3. What:  Materials | Tablet device with the KOKU application (German version), user manual, and safety instructions. | Tablet device for video calls, wrist-worn activity tracker with accompanying tracker application installed on the tablet, and self-designed worksheets for individual barrier identification, goal setting, and action planning, and an activity calendar for additional self-tracking of PA. |
| 4. What:  Procedures | During a one-time home visit, all materials are provided and participants receive an introduction to the tablet and the KOKU application. Exercises are tested with the participants and corrected if necessary to ensure safe execution. Participants are then encouraged to perform the KOKU exercises independently three times per week for 12 weeks, following the app-guided progressive training plan. | PC comprises three structured video-call sessions conducted over the 12-week intervention period. Participants formulate individualized physical activity goals, with the coach assuming a primarily supportive, participant-centered role. Sessions are delivered using a motivational interviewing approach and integrate evidence-based BCTs throughout.  Session 1 focuses on individualized goal setting and action planning, including formulation of SMART activity goals and a specific, measurable action plan.  Session 2 addresses evaluation of the action plan, identification and management of barriers through problem-solving strategies, and integration of social support into the action plan.  Session 3 focuses on reinforcement and maintenance planning, including evaluation of overall progress and strategies to maintain and enhance PA in daily life beyond the intervention period. |
| 5. Who provided | Trained study staff (physiotherapists, sports scientists) provide a one-time introduction during the home visit; subsequent exercise instruction and training guidance are delivered autonomously by the KOKU application. | Trained study staff (physiotherapists, sport scientists) |
| 6. How | KOKU is delivered digitally via a tablet-based application, with exercises performed independently at home following app-guided instructions and progression. | PC is delivered remotely via scheduled tablet-based video calls between the coach and the participant. |
| 7. Where | At participants’ homes | Coaching sessions take place remotely, with participants located at home or another location with a stable internet connection, and coaches located at the study center or another remote location. |
| 8. When and how much | KOKU starts 8±2 weeks post-THA, after completion of the standard rehabilitation phase and the one-time home visit (approximately 60 min). The minimum training duration is 12 weeks; continued use is possible indefinitely.  Dosage: Three sessions per week, each including three exercises, for approximately 15 minutes per session; participants may optionally perform additional exercises beyond the prescribed sessions. If KOKU is not used for two days, the app automatically generates a push notification reminder on the participant’s tablet device.  Intensity: Progressive and individually adapted based on participant feedback and predefined difficulty levels; the number of repetitions is individually determined and self-recorded after each exercise. | PC starts after KOKU initiation. Three video-call sessions of approximately 45-60 minutes.  Session 1 takes place in intervention week 2 (approximately week 10 post-THA), session 2 in intervention weeks 3-4 (approximately weeks 11-12 post-THA), and session 3 in intervention week 12 (approximately week 20 post-THA). |
| 9. Tailoring | KOKU includes 26 different strength and balance exercises of varying difficulty levels (sitting, standing, walking). Exercise progression is individually tailored based on participant feedback regarding perceived safety and ability (“Were you able to perform the exercise without any concern?”) and training frequency. Participants determine the number of repetitions and session frequency within recommended limits (self-paced progression). | PC is highly individualized and guided by a semi-structured manual with specific questions designed to help participants explore themselves. Session content and emphasis are adapted to participants’ individual needs, circumstances, goals, and progress over time. |
| 10. Modifications | N/A | N/A |
| 11. How well:  Planned | Adherence is supported by an in-app progress tracking display when opening KOKU (completed sessions and remaining sessions to reach 12 weeks). Training adherence is assessed after the intervention period at the 6-month postoperative assessment using KOKU usage data recorded by the application and the Exercise Adherence Rating Scale. Acceptability of KOKU is evaluated using the TFA questionnaire and a single global acceptability item. | Fidelity is monitored through documentation by trained staff after each session, including session duration, attendance/completion, and completion of a checklist covering key session topics. Acceptability of PC is evaluated using the TFA questionnaire and a single global acceptability item. |
| 12. How well:  Actual | N/A | N/A |
| Abbreviations: BCT, behavior change technique; KOKU, Keep On Keep Up; N/A, not applicable; PA, physical activity; PC, personal coaching; SMART, specific, measurable, achievable, relevant, and time-bound; THA, total hip arthroplasty; TFA, Theoretical Framework of Acceptability. | | |
